# Partial Visual Loss Affects Self-reports of Hearing Abilities Measured Using a Modified Version of the Speech, Spatial, and Qualities of Hearing Questionnaire

**DOI:** 10.3389/fpsyg.2017.00561

**Published:** 2017-04-12

**Authors:** Andrew J. Kolarik, Rajiv Raman, Brian C. J. Moore, Silvia Cirstea, Sarika Gopalakrishnan, Shahina Pardhan

**Affiliations:** ^1^Vision and Eye Research Unit, Postgraduate Medical Institute, Anglia Ruskin UniversityCambridge, UK; ^2^Department of Psychology, University of CambridgeCambridge, UK; ^3^Centre for the Study of the Senses, Institute of Philosophy, University of LondonLondon, UK; ^4^Shri Bhagwan Mahavir Vitreoretinal Services, Sankara Nethralaya Eye HospitalChennai, India; ^5^Faculty of Low Vision Care, Elite School of OptometryChennai, India

**Keywords:** blindness, auditory perception, spatial hearing, vision loss, speech perception, vision disorders

## Abstract

We assessed how visually impaired (VI) people perceived their own auditory abilities using an established hearing questionnaire, the Speech, Spatial, and Qualities of Hearing Scale (SSQ), that was adapted to make it relevant and applicable to VI individuals by removing references to visual aspects while retaining the meaning of the original questions. The resulting questionnaire, the SSQvi, assessed perceived hearing ability in diverse situations including the ability to follow conversations with multiple speakers, assessing how far away a vehicle is, and the ability to perceptually segregate simultaneous sounds. The SSQvi was administered to 33 VI and 33 normally sighted participants. All participants had normal hearing or mild hearing loss, and all VI participants had some residual visual ability. VI participants gave significantly higher (better) scores than sighted participants for: (i) one speech question, indicating less difficulty in following a conversation that switches from one person to another, (ii) one spatial question, indicating less difficulty in localizing several talkers, (iii) three qualities questions, indicating less difficulty with segregating speech from music, hearing music more clearly, and better speech intelligibility in a car. These findings are consistent with the perceptual enhancement hypothesis, that certain auditory abilities are improved to help compensate for loss of vision, and show that full visual loss is not necessary for perceived changes in auditory ability to occur for a range of auditory situations. For all other questions, scores were not significantly different between the two groups. Questions related to effort, concentration, and ignoring distracting sounds were rated as most difficult for VI participants, as were situations involving divided-attention contexts with multiple streams of speech, following conversations in noise and in echoic environments, judging elevation or distance, and externalizing sounds. The questionnaire has potential clinical applications in assessing the success of clinical interventions and setting more realistic goals for intervention for those with auditory and/or visual losses. The results contribute toward providing benchmark scores for VI individuals.

## Introduction

A large number of studies have demonstrated that full visual loss is often associated with changes in auditory abilities, principally within the spatial domain. For blind people (referred to here as those with full visual loss or light perception only), supra-normal auditory abilities have been reported for a number of spatial tasks, including localizing sounds in azimuth in the periphery (Voss et al., 2004), localization in azimuth using monaural cues only (Lessard et al., 1998; Gougoux et al., 2005; Voss et al., 2008, 2015), and distance discrimination of sounds more than 1 m from the participant, in extrapersonal space (Voss et al., 2004; Kolarik et al., 2013a). However, reduced performance has been reported for other auditory tasks, such as vertical localization in quiet (Lewald, 2002; Voss et al., 2015) and in background noise (Zwiers et al., 2001), and absolute distance judgments in extrapersonal space (Kolarik et al., 2013b, 2017). For reviews, see Collignon et al. (2009), Voss et al. (2010), Kolarik et al. (2014, 2016), Voss (2016). The different pattern of results across different tasks may be accounted for in terms of two hypotheses. The perceptual enhancement hypothesis holds that auditory abilities may be improved for blind individuals, due to substantial experience with and reliance on auditory information (Rice, 1970) and to compensatory processes, such as cortical reorganization, that may increase the efficiency of auditory processing (Voss and Zatorre, 2012). On the other hand, the perceptual deficiency hypothesis suggests that, without visual information to calibrate audition, deficits in auditory abilities occur in blind compared to sighted individuals (Axelrod, 1959; Jones, 1975). Both hypotheses have been proposed in the literature to explain research data and it is possible that both may be valid depending on the exact nature of the task. However, the factors determining which of the two alternatives is more dominant in a given task are not fully understood.

Although there are many psychophysical and neurological studies on the effects of total or near-total loss of vision on auditory spatial abilities, the effects of partial visual loss (referred to here as visual impairment (VI), for participants who have some residual visual ability) are generally under-researched. These abilities, however, are important for people with progressive ocular diseases, who have reduced central or peripheral vision. One of the few studies that have investigated the effect of partial visual loss on hearing ability revealed enhanced auditory localization for participants with one blind eye (Hoover et al., 2012). Enhanced auditory abilities for localization in azimuth have also been shown for myopic (short-sighted) participants when compared to normally sighted participants (Dufour and Gérard, 2000; Després et al., 2005). However, other studies have reported no difference or poorer performance for VI participants relative to sighted controls for localization in azimuth (Lessard et al., 1998), and distance discrimination (Kolarik et al., 2013a), so the overall picture remains unclear. In the current study, we investigated whether partial visual loss affected participants' subjective judgments of their own hearing abilities, as assessed using a questionnaire.

The Speech, Spatial, and Qualities Questionnaire, or SSQ (Gatehouse and Noble, 2004) has been designed to investigate the disabling effects of hearing loss, and is split into three sections addressing different aspects of hearing. The speech section addresses the ability to follow speech in different contexts. The spatial section addresses perception of the distance, direction, and movement of sounds. The qualities section assesses hearing experience such as perceived naturalness and identification of sound sources. The SSQ has been used extensively to investigate self-perceived hearing ability for hearing-impaired participants (Gatehouse and Noble, 2004; Noble and Gatehouse, 2004; Akeroyd et al., 2007), participants wearing hearing aids (Noble and Gatehouse, 2006), and participants with cochlear implants (Summerfield et al., 2006; Spriet et al., 2007; Noble et al., 2008). The SSQ was designed to assess “auditory disability,” i.e., the extent to which auditory abilities limit the performance of activities that are accepted as normal. This is in contrast to “auditory impairment,” which is related to objectively measured functional auditory loss (Agus et al., 2009).

Self-report subjective assessment of hearing abilities has not been examined to date for VI participants. Such an assessment would provide an insight into whether enhancements or deficits of hearing abilities are perceived by those with partial visual losses relative to those with full vision. The aim of this study was to investigate self-reported auditory abilities of VI participants for a wide range of situations and sound stimuli, to explore which conditions are perceived to be easy or difficult compared to normally sighted individuals. Although benchmark SSQ scores have been reported for normally sighted individuals (e.g., Banh et al., 2012), equivalent benchmark scores for VI individuals have not yet been reported, although the results of the studies described above (Dufour and Gérard, 2000; Després et al., 2005; Hoover et al., 2012) suggest that visual loss is likely to affect self-reported hearing abilities. Benchmark scores for VI individuals could be utilized for assessing clinical interventions for such individuals, with or without hearing loss, and for establishing situations in which VI individuals find hearing most challenging. The results could also inform current theories about the effects of visual loss on auditory abilities, by providing for the first time subjective data indicating which auditory abilities are perceived to be improved, in line with the perceptual enhancement hypothesis, and which, if any, are perceived to be poorer, in line with the perceptual deficit hypothesis.

We modified the SSQ for use by VI participants by making minimal changes to make the questionnaire applicable to VI participants while preserving the meaning of the original SSQ questions. The resulting questionnaire, the SSQvi, is intended to be applicable to participants who have partial visual losses or full blindness as well as those who have full vision, and provides subjective measures of abilities and experiences for a wide range of auditory situations. To date, the abilities relating to segregating sounds, recognizing sounds, and the amount of effort needed for listening have not yet been assessed, either experimentally or via questionnaires, for VI participants. The SSQvi allows these abilities to be assessed, and allows identification of the auditory situations in which VI participants have the most difficulty in everyday life.

## Materials and methods

### Participants

Two groups of participants completed the SSQvi: a VI group (*n* = 33, 7 females, mean age 44 years, range 35–53 years), and a normally sighted group (*n* = 33, 24 females, mean age 41 years, range 36–50 years). A *t*-test showed no significant difference in age between the VI group and normally sighted control group [*t*_(64)_ = 1.97, ns]. Participants in the VI group were assigned to categories 1–3 of visual loss as defined by the World Health Organization (1989): Category 1 is moderate visual impairment (distance visual acuity equal to or better than 6/60, but worse than 6/18, *n* = 12), category 2 is severe visual impairment (distance visual acuity equal to or better than 3/60, but worse than 6/60, *n* = 19), and category 3 is blindness, with remaining vision (distance visual acuity equal to or better than 1/60, but worse than 3/60, *n* = 2). The criteria for VI, as used in the current paper, were chosen to distinguish participants with remaining vision from those with “total” blindness, as described in previous papers (Voss et al., 2008; Kolarik et al., 2013a,c). Participants categorized as totally blind had light perception only, or no light perception, as defined by WHO criteria categories 4 and 5, respectively. Table 1 gives the distribution of VI participants according to the age when their visual loss started and the distribution of participants according to the duration of visual loss. These variables may be relevant to the development of cortical reorganization. For people who are fully blind, enhanced perceptual abilities have been reported to arise following cortical reorganization, and the degree of enhancement is dependent on the age of onset of blindness (Voss et al., 2008). It is not yet clear whether age of onset plays a role in cortical reorganization for VI participants. For participants with one eye, Hoover et al. (2012) found no correlation between age of visual loss by enucleation and sound localization performance, but they tested a relatively small sample (*n* = 10) with a limited range of time since enucleation (18–39 months). Table 2 gives the causes of visual loss. All normally sighted participants had normal or corrected-to-normal vision.

**Table 1:** **Summary of the number of VI participants whose visual loss started in each age range (e.g., there were 2 participants whose visual loss started when they were between 0 and 5 years old), and the number of participants in each range of duration of visual loss (e.g., there were 12 participants whose duration of visual loss was between 0 and 5 years)**.

**Range (years)**	**Age: number of participants**	**Duration: number of participants**
0–5	2	12
6–10	3	9
11–15	3	0
16–20	0	3
21–25	0	1
26–30	4	3
31–35	5	1
36–40	8	2
41–45	5	1
46–50	2	1
51–55	1	0
Total	33	33

**Table 2:** **Summary of causes of visual loss and number of VI participants with each condition, and pure-tone average (PTA) thresholds for the better ear**.

**Cause of vision loss**	**PTA**	**Visual acuity**
OU: Healed Choroiditis	21	OD: 6/60, OS: 3/60
OU: Healed Choroiditis	24	OD: 1/30, OS: 6/38
OU: Retinochoroidal Coloboma	25	OD: 2/30, OS: 6/76
OU: Retinochoroidal Coloboma	28	OD: 6/60, OS: 6/60
OU: Heredo Macular Degeneration	18	OD: 6/19, OS:1/60
OU: Retinal Detachment	40	OD: CFCF, OS: 6/76
OD: Retinal Detachment Surgery, OS: Old Retinal detachment	38	OD: 6/48, OS: No PL
OU: Bulls Eye Maculopathy	11	OD: 6/15, OS: 6/15
OU: Foveal Thinning	19	OD: 6/38, OS: 6/38
OU: Optic Nerve damage	24	OD: 6/9, OS: 6/9
OU: Glaucoma	14	OD: PL+, OS: 6/24
OU: Optic Neuropathy	29	OD: 6/38, OS: 6/48
OU: Retinal Pigment Epithelium Atrophy with Overlying Thinned Retina	14	OD: 6/60, OS: 6/60
OU: Proliferative Diabetic Retinopathy	29	OD: 6/36, OS: CFCF
OU: Proliferative Diabetic Retinopathy	30	OD: HM, OS: 6/60
OU: Stargardt's Disease with Foveal Atrophy	33	OD: 6/76, OS: 6/76
OU: Proliferative Diabetic Retinopathy, Pale Disc	29	OD: 6/60, OS: 6/60
OU: Cone-Rod Dystrophy	28	OD: 6/30, OS: 6/19
OU: Retinitis Pigmentosa, OS: Total Cataract	25	OD: 6/60, OS: PL+
OU: Temporal Pallor	31	OD: 6/18, OS: 6/24
OU: Macular Scar	24	OD: 6/76, OS: 6/76
OU: Macular Scar	39	OD: 6/90, OS: 2/38
OU: Non-Arteritic Anterior Ischemic Optic Neuropathy	34	OD: 6/18, OS: 6/18
OU: Glaucoma, OD: Aphakia	34	OD: 6/36, OS: No PL
OU: Glaucoma, Posterior Staphyloma	17	OD: 6/60, OS: 6/24
OD: Corneal Opacity, OS: S/p Retinal Detachment Sx	31	OD: HM+, OS: 6/76
OU: Retinitis Pigmentosa, Rubella Retinopathy	29	OD:6/30, OS: 6/19
OU: Retinitis Pigmentosa	25	OD: 6/76, OS: 6/76
OU: Retinitis Pigmentosa	24	OD: 2/60, OS: 2/60
OU: Retinitis Pigmentosa	26	OD: 6/78, OS: 6/78
OU: Retinitis Pigmentosa	28	OD: 1/30, OS: 6/76 FOV <5 degrees
OU: Retinitis Pigmentosa	25	OD: 6/38, OS: 6/24
OU: Retinitis Pigmentosa	20	OD: 6/38, OS: 6/24

*OD, (oculus dexter): right eye; OS, (oculus sinister): left eye; OU, (oculi uterque): both eyes; PL+, Perception of light; FOV, field of vision; CFCF, counting fingers close to face; HM, Hand movements close to face; S/p, Status post; Sx, Surgery*.

Hearing status was categorized as proposed by Goodman (1965). Based on the measured pure-tone average (PTA) better ear thresholds across the frequencies 0.5, 1, 2, and 4 kHz, all participants (both VI and sighted controls) had normal hearing (PTA 10–26 dB) or mild hearing loss (PTA 27–40 dB). A *t*-test showed no significant difference in PTA between the VI group and normally-sighted control group [*t*_(64)_ = 1.50, ns]. The thresholds were consistent with the overall age range of the participants (35–53 years), some of whom had moderate elevations in audiometric thresholds relative to young individuals (Brant and Fozard, 1990; Wiley et al., 2008). For the VI group and sighted controls, respectively, the mean number of years of education completed was 10.6 (*SD* = 7.8) and 16.4 (*SD* = 4.1) years. The number of years of education completed was significantly greater for the sighted control group compared to the VI group [*t*_(64)_ = 3.74, *p* < 0.01]. However, care was taken to ensure that all participants understood the task clearly.

The research followed the tenets of the Declaration of Helsinki, and informed consent was obtained from all participants after the nature and possible consequences of the study were explained. The research was approved by the Anglia Ruskin Research Ethics Panel and by the Sankara Nethralaya Ethics Panel.

### Data collection

Data were collected from participants in Chennai, India at the Sankara Nethralaya Eye Hospital, where they underwent a visual assessment and audiometric testing. All the participants who were referred to the Low Vision Care clinic based on their visual acuity criteria between April 2015 and December 2015 were included. The participants were tested using the Indian languages of Tamil or Hindi, using a translated version of the SSQvi. The translated SSQvi was back-translated by another translator for both languages to confirm consistency with the original SSQvi. As previous work has shown that the method of SSQ administration did not systematically affect scores (Singh and Pichora-Fuller, 2010), the SSQvi was administered to participants either by interview or by self-administration. All participants except two sighted participants were tested by interview. In interviews, participants responded verbally, and responses were recorded by the experimenter.

### Questionnaire design

The SSQvi was modified from the SSQ questionnaire version 5.6. The Appendix contains all SSQvi questions. Modifications were limited to minimal changes required to make the questionnaire applicable to participants with visual losses whilst preserving the meaning of the original questions. Questions without any visual aspect were not altered. Nine questions that referred to the visibility of sound sources were changed by removing the visual aspect of the question (SSQ 5.6 speech questions: 3, 4; spatial: 1, 2, 3, 6, 7, 15, and 16). For example, for speech question 3, the SSQ question was modified from “You are in a group of about five people, sitting round a table. It is an otherwise quiet place. You can see everyone else in the group. Can you follow the conversation?” to “You are in a group of about five people, sitting round a table. It is an otherwise quiet place. Can you follow the conversation?”

For the questions with the visual aspect removed, people may have answered the rephrased questions with regard to their hearing only, or with regard to the combined use of hearing and vision, including remaining vision for VI participants. To address the potential role of visual cues, responses to the 9 modified SSQvi questions were compared to those for the 9 original SSQ questions for a group of normally sighted participants (*n* = 39, 26 females, mean age 40 years, range 35–50 years). SSQ and SSQvi responses were obtained with an interval between the two of at least 12 months. Table 3 shows mean SSQ and SSQvi scores and standard errors for each of the 9 questions. For the two speech items, removing the visual aspect (where SSQ questions stated that the targets could be seen) had only a very small influence on responses, suggesting that vision did not markedly influence responses to the modified speech questions. However, larger differences were obtained for the modified spatial questions. SSQvi questions where the role of visual information was ambiguous received higher scores, and SSQ questions which stated that the target could not be seen received lower scores, suggesting a potential role for vision in the modified spatial questions (see the Discussion for implications for VI and fully blind participants).

**Table 3:** **Means and standard errors of SSQ and SSQvi scores for normally sighted participants, for the nine SSQ questions that were changed by removing the visual aspect of the question in the SSQvi**.

	**Question description**	**Question number: SSQ/SSQvi**	**SSQ Mean**	**SSQvi Mean**	**SSQ Standard error**	**SSQvi Standard error**
Speech	Having conversation with five people in quiet	3/3	9.41	9.69	0.15	0.16
	Having conversation with five people in noise	4/4	9.18	9.36	0.20	0.21
Spatial	Locate lawnmower	1/14	8.85	9.82	0.23	0.09
	Locate speaker around a table	2/15	8.87	9.85	0.28	0.07
	Localize a talker to left to right	3/16	9.31	9.90	0.17	0.06
	Locate dog barking	6/19	9.23	9.79	0.17	0.09
	Locate vehicle from footpath	7/20	9.31	9.92	0.19	0.04
	Sounds closer than expected	15/28	8.44	9.64	0.32	0.12
	Sounds farther than expected	16/29	8.05	9.64	0.34	0.16

*Equivalent SSQ and SSQvi question numbers are given (e.g., Spatial item 1 in the SSQ is equivalent to SSQvi question 14)*.

SSQ Speech question 6 was removed as it is identical to speech question 4, except that the people in the scenario can be seen. SSQ Qualities question 16 was removed as it refers to driving, and as a result of this removal a minor modification was made to SSQ qualities question 17 (question 46 in the SSQvi) to specify that the listener was in a car. Qualities question 15 from the original SSQ, which concerned turning off a hearing aid or cochlear implant, was not included, following its exclusion in SSQ 5.6. The final questionnaire contained 47 questions. To avoid overlapping question numbers between categories, the questions in the Spatial and Qualities sections were renumbered, so that all questions were numbered from 1 to 47. The visual response scale was removed and participants were asked to give a single number between 0 and 10 for each question, either verbally or as a written response.

A score of 10 represented a perfect ability and 0 indicated that the listener was unable to do or experience what was described. Although the instructions made it clear that any question could be deemed inapplicable, all participants provided a response for all questions. All gave only integer scores.

## Results

Table 4 shows mean scores and standard errors for each of the SSQvi questions for the VI participants, and Table 5 shows corresponding data for the sighted controls. Questions are ordered for each section according to the mean scores they received. For both groups, mean scores were 8.7 or higher, indicating that hearing abilities were perceived to be good overall. Scores for the speech questions were generally lower than those for the other sections. For both groups, speech questions that were rated as most difficult related to divided-attention contexts where multiple streams of speech had to be followed at the same time. For the spatial section, questions related to distance and vertical location were rated as hardest by both groups, and VI participants also rated the question related to externalization of sounds as one of the most difficult. For the section assessing qualities of sound for the VI group, questions involving effort, ability to ignore competing sounds, and concentration were rated as hardest. For the sighted group, questions relating to identifying instruments in music, understanding a conversation in a car, and ignoring competing sounds were rated as hardest.

**Table 4:** **Means and standard errors of scores for the VI participants for speech, spatial, and qualities questions**.

	**Question description**	**Question no**.	**Mean**	**Standard error**
Speech	Having conversation with five people in noise	4	8.76	0.34
	Follow one person speaking and telephone at same time	13	8.88	0.41
	Talk with one person and follow TV	9	8.94	0.35
	Having conversation in echoic environment	6	9.06	0.30
	Ignore interfering voice of different pitch	8	9.36	0.25
	Talking with one person in continuous background noise	5	9.42	0.24
	Follow one conversation when many people talking	10	9.45	0.22
	Ignore interfering voice of same pitch	7	9.55	0.19
	Talking with one person with TV on	1	9.76	0.15
	Follow conversations without missing start of new talker	11	9.76	0.14
	Talking with one person in quiet room	2	9.79	0.16
	Having conversation with five people in quiet	3	9.85	0.12
	Have conversation on telephone	12	9.97	0.03
Spatial	Locate above or below on stairwell	18	9.24	0.32
	Externalization of sounds	27	9.39	0.36
	Judge distance of vehicle	22	9.45	0.34
	Judge distance from footsteps or voice	21	9.52	0.29
	Locate vehicle from footpath	20	9.55	0.25
	Identify lateral movement from voice or footsteps	24	9.61	0.28
	Identify whether a vehicle is approaching or receding	26	9.64	0.31
	Identify lateral movement of vehicle	23	9.64	0.22
	Locate lawnmower	14	9.67	0.21
	Sounds in expected location	30	9.70	0.21
	Sounds closer than expected	28	9.70	0.30
	Identify approach or recede from voice or footsteps	25	9.79	0.21
	Locate a door slam in unfamiliar house	17	9.82	0.15
	Locate dog barking	19	9.82	0.15
	Sounds further than expected	29	9.85	0.15
	Locate speaker around a table	15	10.00	0.00
	Localize a talker to left to right	16	10.00	0.00
Qualities	Effort of conversation	45	9.18	0.44
	Ability to ignore competing sounds	47	9.39	0.36
	Need to concentrate when listening	44	9.48	0.34
	Distinguish different sounds	36	9.67	0.30
	Distinguish familiar music	35	9.67	0.30
	Separation of two sounds	31	9.85	0.15
	Naturalness of everyday sounds	41	9.85	0.15
	Understand when car passenger	46	9.85	0.15
	Identify instruments in music	37	9.91	0.09
	Clarity of everyday sounds	39	9.97	0.03
	Judging mood by voice	43	9.97	0.03
	Music and voice as separate objects	33	10.00	0.00
	Sounds appearing jumbled	32	10.00	0.00
	Naturalness of own voice	42	10.00	0.00
	Naturalness of other voices	40	10.00	0.00
	Identify different people by voice	34	10.00	0.00
	Naturalness of music	38	10.00	0.00

*Questions are ordered from lowest scores (most difficult) to highest scores (easiest), for each section*.

**Table 5:** **As for Table 4, but for sighted control participants**.

	**Question description**	**Question no**.	**Mean**	**Standard error**
Speech	Talk with one person and follow TV	9	8.73	0.25
	Follow conversations without missing start of new talker	11	8.88	0.25
	Follow one person speaking and telephone at same time	13	9.18	0.23
	Ignore interfering voice of different pitch	8	9.18	0.20
	Follow one conversation when many people talking	10	9.24	0.21
	Ignore interfering voice of same pitch	7	9.27	0.19
	Having conversation in echoic environment	6	9.27	0.22
	Having conversation with five people in noise	4	9.33	0.22
	Talking with one person with TV on	1	9.58	0.22
	Talking with one person in continuous background noise	5	9.61	0.20
	Talking with one person in quiet room	2	9.70	0.14
	Having conversation with five people in quiet	3	9.73	0.16
	Have conversation on telephone	12	9.76	0.10
Spatial	Locate above or below on stairwell	18	8.94	0.23
	Judge distance from footsteps or voice	21	9.06	0.22
	Judge distance of vehicle	22	9.27	0.20
	Judge lateral movement from voice or footsteps	24	9.39	0.20
	Judge approach or recede from voice or footsteps	25	9.42	0.19
	Sounds closer than expected	28	9.42	0.17
	Sounds in expected location	30	9.45	0.18
	Externalization of sounds	27	9.45	0.14
	Locate a door slam in unfamiliar house	17	9.48	0.15
	Locate dog barking	19	9.55	0.16
	Judge lateral movement of vehicle	23	9.58	0.15
	Sounds further than expected	29	9.61	0.14
	Judge whether a vehicle is approaching or receding	26	9.61	0.14
	Locate vehicle from footpath	20	9.61	0.12
	Locate each speaker around a table	15	9.64	0.13
	Locate lawnmower	14	9.67	0.11
	Locate a talker to left to right	16	9.79	0.09
Qualities	Identify instruments in music	37	9.39	0.21
	Understand when passenger in a car	46	9.42	0.20
	Ability to ignore competing sounds	47	9.55	0.12
	Effort of conversation	45	9.64	0.16
	Music and voice as separate objects	33	9.70	0.08
	Naturalness of music	38	9.70	0.10
	Distinguish different sounds	36	9.73	0.10
	Sounds appearing jumbled	32	9.76	0.12
	Need to concentrate when listening	44	9.79	0.10
	Distinguish familiar music	35	9.82	0.09
	Judging mood by voice	43	9.82	0.08
	Clarity of everyday sounds	39	9.82	0.08
	Separation of two sounds	31	9.88	0.06
	Naturalness of other voices	40	9.88	0.06
	Identify different people by voice	34	9.88	0.06
	Naturalness of own voice	42	9.88	0.06
	Naturalness of everyday sounds	41	9.97	0.03

Descriptive information regarding individual differences between participants is given in Tables 6–10. Summaries are given of mean scores across all questions of the speech, spatial and qualities sections of the SSQvi, for VI participants in each WHO category (Table 6), and for VI and normally sighted participants with normal hearing or mild hearing loss (Table 7). Statistical analysis was not possible given the small numbers of participants in each subgroup. Participants with severe visual impairment scored more poorly than those with moderate visual impairment or blindness with remaining vision (Table 6). For both VI and sighted groups, overall SSQvi scores were either similar to or, surprisingly, slightly higher for those with mild hearing loss than for those with normal hearing. However, the standard deviations of the scores were large, so there was considerable overlap between scores for those with mild hearing loss and those with normal hearing (Table 7). Table 8 shows that age of onset of visual loss did not generally affect scores. Table 9 shows that overall SSQvi scores were lowest for VI participants with a duration of visual loss between 6 and 10 years. However, this group also showed the most variability in their responses, so again scores overlapped considerably across groups with different durations of visual loss. Although there were no substantial changes in average scores across different age groups, the older participants (ages 41–55 years) generally scored highest overall (Table 10).

**Table 6:** **Summary of mean scores across all questions of the speech, spatial and qualities sections of the SSQvi, for VI participants in each WHO category**.

**WHO category**	**Number of participants**	**Speech (mean, *SD*)**	**Spatial (mean, *SD*)**	**Qualities (mean, *SD*)**
1	12	9.63, 0.57	9.98, 0.08	9.99, 0.05
2	19	9.27, 1.00	9.44, 1.27	9.68, 0.74
3	2	9.73, 0.38	10.00, 0.00	10.00, 0.00

*Category 1 is moderate visual impairment, category 2 is severe visual impairment, and category 3 is blindness, with remaining vision*.

**Table 7:** **Summary of mean scores across all questions of the speech, spatial, and qualities sections of the SSQvi, for VI and normally sighted control participants with normal hearing (PTA 10-26 dB) or mild hearing loss (PTA 27-40 dB)**.

**Group**	**Hearing status**	**Number of participants**	**Speech (mean, *SD*)**	**Spatial (mean, *SD*)**	**Qualities (mean, *SD*)**
VI	Normal	17	9.32, 0.89	9.61, 1.12	9.78, 0.65
	Mild loss	16	9.54, 0.81	9.73, 0.86	9.85, 0.51
Sighted	Normal	26	9.32, 0.67	9.39, 0.65	9.69, 0.34
	Mild loss	7	9.44, 1.14	9.75, 0.67	9.92, 0.22

**Table 8:** **Summary of mean scores across all questions of the speech, spatial, and qualities sections of the SSQvi, for VI participants, categorized according to the age of onset of their visual loss**.

**Age of onset (years)**	**Number of participants**	**Speech (mean, *SD*)**	**Spatial (mean, *SD*)**	**Qualities (mean, *SD*)**
0–20	9	9.62, 0.82	9.77, 0.58	9.92, 0.20
21–40	15	9.33, 0.95	9.42, 1.38	9.65, 0.83
41–60	9	9.40, 0.73	9.98, 0.04	9.97, 0.08

**Table 9:** **Summary of mean scores across all questions of the speech, spatial, and qualities sections of the SSQvi, for VI participants, categorized according to the duration of their visual loss**.

**Duration (years)**	**Number of participants**	**Speech (mean, *SD*)**	**Spatial (mean, *SD*)**	**Qualities (mean, *SD*)**
0–5	12	9.36, 0.83	9.68, 0.99	9.78, 0.59
6–10	9	9.20, 1.03	9.44, 1.45	9.68, 0.88
11–50	12	9.67, 0.71	9.83, 0.51	9.94, 0.17

**Table 10:** **Summary of mean scores across all questions of the speech, spatial, and qualities sections of the SSQvi, for VI and normally sighted control participants subdivided by age**.

**Group**	**Age range (years)**	**Number of participants**	**Speech (mean, *SD*)**	**Spatial (mean, *SD*)**	**Qualities (mean, *SD*)**
VI	30–40	14	9.38, 0.91	9.54, 1.23	9.75, 0.71
	41–55	19	9.46, 0.82	9.76, 0.79	9.86, 0.47
Sighted	30–40	18	9.26, 0.78	9.35, 0.71	9.72, 0.33
	41–55	15	9.44, 0.78	9.60, 0.59	9.77, 0.34

**Figure 1:**
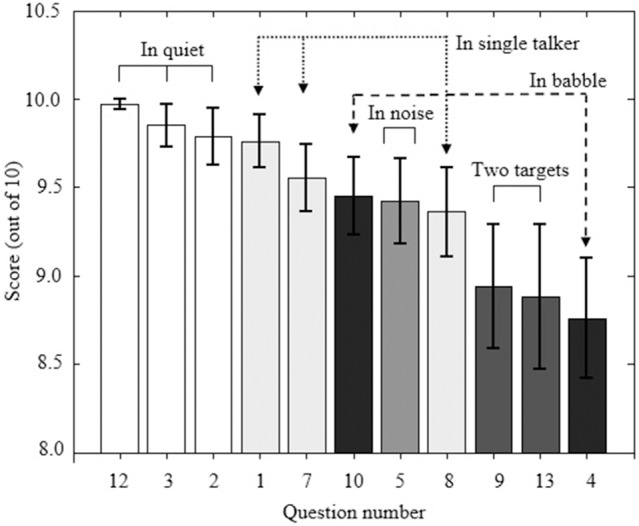
**Mean SSQvi speech scores for VI participants**. Questions are reverse-ordered by mean score. Questions are labeled according to the configuration of target speech and competing speech using the nomenclature of Agus et al. (2009). Error bars represent ±1 standard error of the mean.

**Figure 2:**
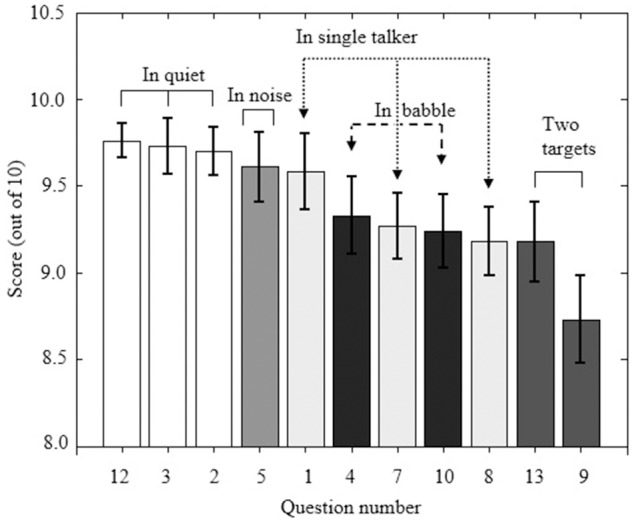
**As Figure 1, but for sighted controls**.

The SSQvi speech questions can be grouped according to the configuration of the target speech and the competing speech, following Agus et al. (2009), providing information regarding which configurations were rated as most difficult by the VI and the sighted participants. Agus et al. categorized speech situations as: following two targets at the same time, multi-talker babble, a single talker, speaking in noise, and speaking in quiet. Questions 6 and 11 are not categorized. Figure 1 shows ratings for VI participants reverse-ordered by mean score for questions that fell within the categories defined by Agus et al. (2009, compare to their Figure 1). Values are the same as reported in Table 4. The most difficult situations involved following two targets and following a conversation in a busy restaurant (question 4). Following speech in noise or with a single competing talker was perceived to be considerably easier, as was following a conversation in competing babble (question 10). Figure 2 shows ratings for speech questions reverse-ordered by mean score for the sighted controls. Values are the same as reported in Table 5. For sighted controls, the most difficult situation involved following two targets (talking with one person and following the TV—question 9). Following speech in babble, with a single competing talker, or in noise was perceived to be easier.

**Figure 3:**
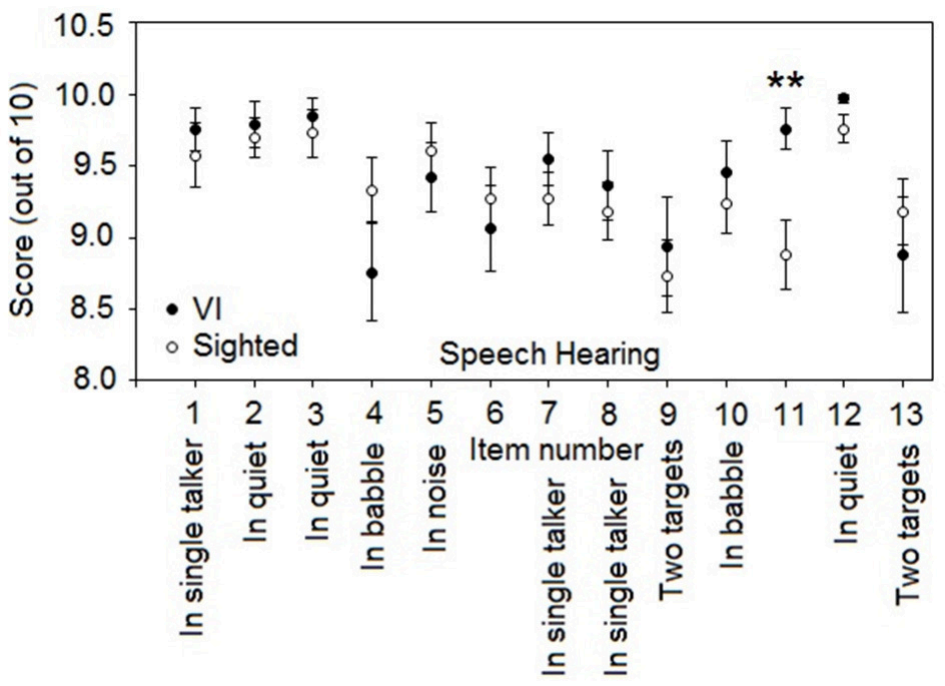
**Mean SSQvi scores for questions from the speech section, for VI participants (closed circles) and normally sighted participants (open circles)**. Values for VI and sighted participants are the same as reported in Tables 4, 5, respectively. Error bars represent ±1 standard error of the mean and are not shown when smaller than the symbol size. Speech questions are labeled according to the nomenclature of Agus et al. (2009). Questions 6 and 11 are not categorized. Here and in subsequent figures, significant differences are shown by asterisks: ^**^p < 0.01.

Figure 3 compares mean SSQvi scores for questions from the speech section, for sighted and VI participants. The significance of differences across the two groups was assessed using Mann-Whitney *U*-tests performed using Bonferroni correction for multiple comparisons. Scores were significantly higher for the VI participants, indicating *less* difficulty, for one of the speech questions: question 11—“You are with a group and the conversation switches from one person to another. Can you easily follow the conversation without missing the start of what each new speaker is saying?” (*U* = 334.5, *p* < 0.01, *r* = 0.42). In general, scores for the VI participants were similar to or better than scores for the sighted participants. Mild hearing loss for some of the participants is unlikely to be responsible for the significant differences in average scores, as PTA was not significantly different between the VI group and normally-sighted control group (see Participants section).

**Figure 4:**
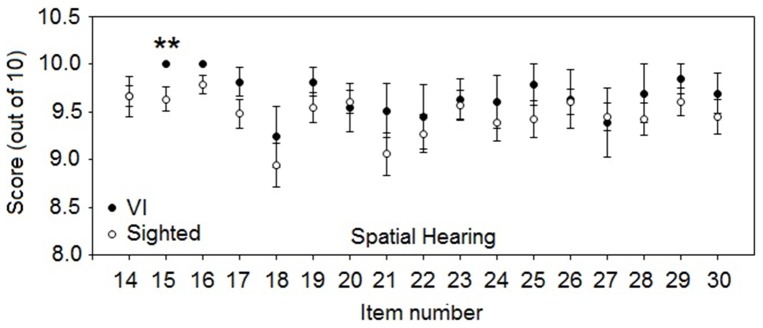
**As for Figure 3, but for mean SSQvi scores for spatial questions**. ^**^p < 0.01.

Scores were significantly higher for VI participants for one of the spatial questions (Figure 4): question 15—“You are sitting around a table or at a meeting with several people. Can you tell where any person is as soon as they start speaking?” (*U* = 412.5, *p* < 0.01, *r* = 0.37). Spatial question scores for VI participants were generally similar to or better than for sighted participants.

**Figure 5:**
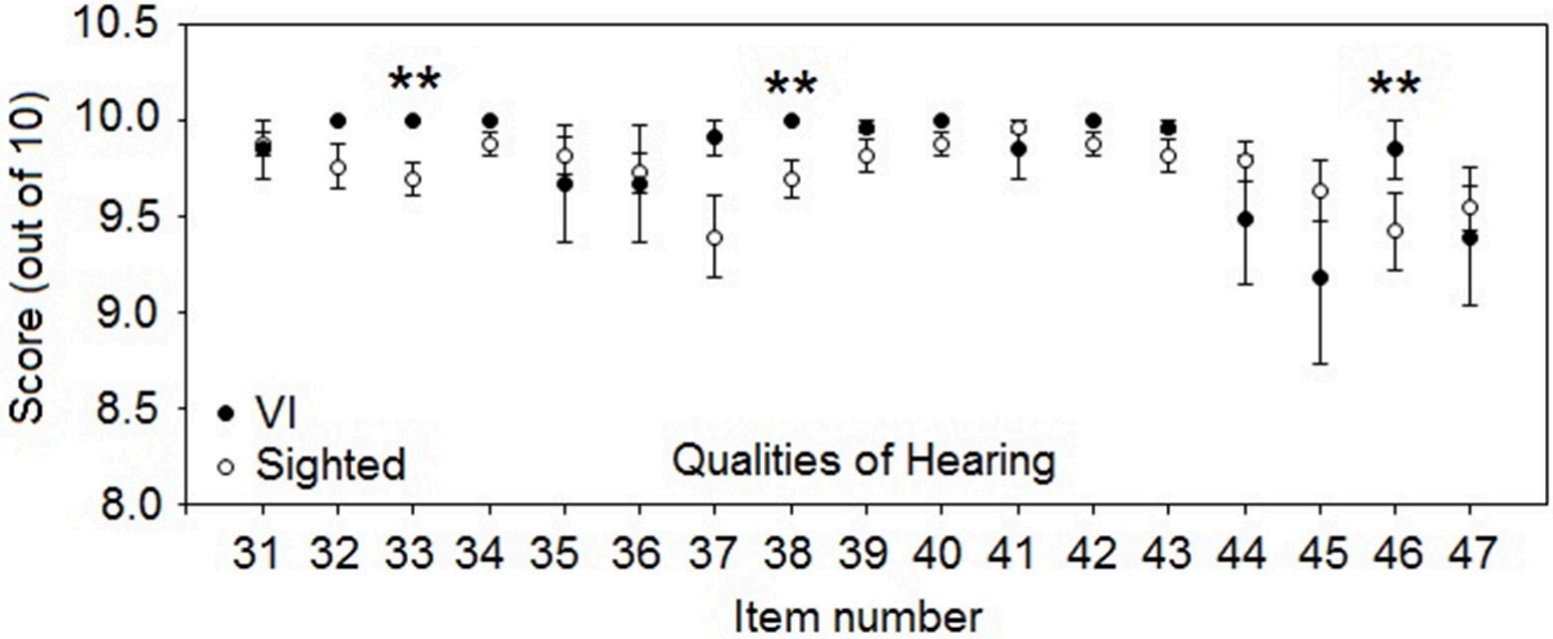
**As for Figure 3, but for mean SSQvi scores for qualities questions**. ^**^p < 0.01.

Scores were significantly higher for VI participants for three of the qualities questions (Figure 5): question 33—“You are in a room and there is music on the radio. Someone else in the room is talking. Can you hear the voice as something separate from the music?” (*U* = 379.5, *p* < 0.01, *r* = 0.42)—, question 38—“When you listen to music, does it sound clear and natural?” (*U* = 412.5, *p* < 0.01, *r* = 0.37)—, and question 46—“When you are a passenger in a car can you easily hear what the driver is saying sitting alongside you?” (*U* = 384.5, *p* < 0.01, *r* = 0.38). No significant differences were found for any other questions. As for the other sections, scores for the VI participants were generally similar to or better than for the sighted participants.

## Discussion

There were three main findings: (1) VI participants gave significantly higher scores than sighted participants for one speech situation involving switching between target talkers; (2) VI participants gave significantly higher scores than sighted participants for one spatial situation, indicating less difficulty in localizing talkers; (3) VI participants gave significantly higher scores than sighted participants for three qualities situations, demonstrating less difficulty in segregating speech from music, hearing music clearly, and understanding speech inside a car. The self-report finding for the spatial question agrees with previously reported objective evidence for partially sighted participants, showing enhanced localization in azimuth (Dufour and Gérard, 2000; Després et al., 2005; Hoover et al., 2012). The SSQvi responses show that VI participants rate their own hearing more highly than do control participants for a number of everyday life auditory abilities, consistent with the perceptual enhancement hypothesis. For all other questions, scores for VI participants were not significantly different to those for sighted controls.

For some SSQvi questions, scores were high for both groups, and ceiling effects may have contributed to the lack of differences between the VI and sighted participants (such as in the qualities of hearing section). As previously highlighted (Voss et al., 2004), ceiling effects may also have affected the results of several previous studies using objective assessments to compare auditory performance for sighted and blind participants, where sighted controls and groups with visual loss showed high performance, precluding any group from performing significantly better than the others (Lessard et al., 1998; Röder et al., 1999; Voss et al., 2004).

### Comparison with previous literature

Localization in azimuth was assessed by 9 of the spatial questions: questions 14, 15, 16, 17, and 19 assessed localization in azimuth for sounds that were static or nearly so, questions 20, 23, and 24 assessed the ability to judge the direction or the direction of movement of dynamic sounds, and question 30 assessed localization in general. Scores were significantly higher for VI participants for spatial question 15. This is consistent with enhanced auditory localization in azimuth for static sounds, as reported in several objective studies of partially sighted participants who had one blind eye (Hoover et al., 2012), or for myopic participants (Dufour and Gérard, 2000; Després et al., 2005). However, one objective study found that VI participants with residual peripheral vision localized sounds less accurately than sighted or totally blind participants (Lessard et al., 1998). Why worse performance for VI participants was found in this study but not in other studies is unclear. However, only three VI participants were tested, and the authors noted that they showed abnormal orienting behaviors such as turning their head toward the source of the experimenter's voice or a test sound so as to make it visible within their remaining visual field, and this may have contributed to lower performance.

Distance perception was assessed by seven of the spatial questions. As described by Akeroyd et al. (2007) for the SSQ, SSQvi spatial questions 28 and 29 assess distance perception in general, questions 21, 22, 25, and 26 assess perception of the distance or changes in distance of dynamic sounds, and question 30 assesses localization in general. There were no significant differences between VI and sighted participants for any of these questions. Consistent with this, Kolarik et al. (2013a) found no difference in auditory distance discrimination between a partially sighted group and a normally sighted group. In contrast, for blind people, objectively measured *absolute distance perception* is poorer than for sighted controls (Kolarik et al., 2013b, 2017), while objectively measured *discrimination of distance* is better than for sighted controls (Voss et al., 2004; Kolarik et al., 2013b). However, one study reported poorer discrimination of distance by blind individuals compared to sighted controls (Cappagli et al., 2015).

For our sighted controls with normal or near-normal-hearing, SSQvi scores were either similar to those for normally hearing young participants tested using the original SSQ by Banh et al. (2012) (e.g., Speech question 2: mean score 9.9 for Banh et al. 9.7 for the current study), or higher for some questions (e.g., Speech question 4: mean score 8.4 for Banh et al. 9.3 for the current study). There are a number of possible reasons for the differences across the studies. These include differences in perceived hearing ability across different countries (India vs. Canada), modification of some questions in the SSQ to remove the visual element in the SSQvi, and differences in visual status (not reported by Banh et al. but assumed to be normal or corrected, as for the current study). Further testing using the SSQvi in other countries with sighted and VI populations would allow the origin of the differences across studies to be clarified. However, as both VI participants and controls in the current study were tested with the same questionnaire and in the same country, it seems reasonable to assume that the observed differences between the groups were due to differences in visual status.

The VI participants rated their own hearing more highly than sighted controls for some but not all of the SSQvi questions. This is consistent with the literature reviewed in the introduction showing that visual loss is associated with superior abilities for some auditory tasks, but not for others. The spatial question for which the VI participants gave significantly higher scores than the control participants addressed judgments of source azimuth, consistent with the finding that VI participants have usually been found to be more accurate than normally sighted participants in judging azimuth (Dufour and Gérard, 2000; Després et al., 2005; Hoover et al., 2012), although one study did not find this to be the case (Lessard et al., 1998). For the single spatial question that addressed judgment of elevation (question 18), the VI group gave a similar mean score to the sighted group. We are not aware of any performance measures of vertical localization comparing VI and sighted groups. For the two questions assessing distance perception (spatial questions 21 and 22), the VI group gave similar mean scores to the sighted group, consistent with the finding of no significant difference in distance discrimination for VI and sighted participants (Kolarik et al., 2013a).

On average, SSQvi scores for the normally sighted and VI participants were higher than those for hearing-impaired listeners obtained using the original SSQ (Gatehouse and Noble, 2004), as would be expected given that the groups in the current study had normal hearing or mild hearing losses. Gatehouse and Noble (2004) reported mean scores ranging from 7.1 to 2.5 for the speech section of the SSQ, from 7.5 to 4.2 for the spatial section, and from 8.3 to 3.7 for the qualities section. In the current study, SSQvi average scores for normally sighted participants ranged from: 9.8 to 8.7 for the speech section, 9.8 to 8.9 for the spatial section, and 10.0 to 9.4 for the qualities section. SSQvi average scores for the VI participants ranged from: 10.0 to 8.8 for the speech section, 10.0 to 9.2 for the spatial section, and 10.0 to 9.2 for the qualities section. Although the visual status of the participants in the study of Gatehouse and Noble (2004) was not reported, it is likely that some of their participants had visual loss that may have affected the scores, as the average participant age was 71 years, and visual as well as auditory abilities often decline with age (Simon and Levitt, 2007). As discussed below, the current findings contribute toward setting a benchmark for scores for those with visual loss that can be compared to scores for people with both visual and auditory losses.

### Future directions: comparison to objective hearing abilities for VI individuals and effects of severity and duration of visual loss

The SSQvi can potentially be used regardless of the visual or auditory status of the participants. To check that the SSQvi did not pose problems for hearing-impaired people or people with dual visual and auditory losses, the SSQvi was administered to three normally sighted hearing-impaired people and two people with dual losses (data not reported). All answered all questions without any difficulty. Hence, the SSQvi is a tool that can be used in future studies of participants with different degrees of visual and/or auditory loss to assess whether self-reported perceptions match objective abilities tested under controlled laboratory conditions. Should this occur, it would suggest that supra-normal auditory abilities measured in the laboratory, when they occur, extend to situations in real life. Supporting this idea, Akeroyd et al. (2007) found significant correlations between experimental measures of distance perception and data from SSQ questions relating to distance perception for older hearing-impaired participants.

As mentioned above, relatively few studies have tested auditory abilities for those with partial visual loss (in particular for those with central or peripheral visual field deficits), and we are not aware of any studies that have assessed auditory abilities for people with dual loss. Furthermore, although improved spatial abilities in early-onset blind individuals are usually associated with cortical plasticity, whereby the visual cortex is recruited for performance of spatial hearing tasks (for reviews, see Collignon et al., 2009; Voss and Zatorre, 2012), evidence is lacking regarding whether such recruitment occurs for those with partial visual loss. Studies of whether and how this occurs in partially sighted individuals could make use of the SSQvi, as well as objective measures of performance and imaging data, to shed light on the conditions required for cortical reorganization to occur and to examine the relationships between subjective and objective performance and the degree or cortical reorganization.

Although trends toward lower scores for VI than for sighted participants occurred for several speech questions (see below), the differences were not significant. It is perhaps surprising that scores were not significantly lower for some speech questions for the VI participants, since the sighted participants would have had the full benefit of visual information, whereas the VI participants would not have. It is well-known that, in difficult listening situations, speech perception for sighted people can be enhanced by visual information from the face of the talker (Sumby and Pollack, 1954). Many normally hearing people are unaware that they use visual information in such situations, even though objective performance is substantially enhanced by the use of visual information (called lip-reading or speech reading). Also, face perception, especially perception of facial expression, assists with verbal communication (Haxby et al., 2000) and this would be less available for VI participants. Several of the SSQvi questions assess the ability to understand speech when background sounds are present, but the questions do not specifically indicate whether or not visual cues are available (e.g., questions 3, 4, 5, and 7–11). It seems likely that the sighted participants answered these questions in relation to their everyday experience, which would include the use of visual information. Trends toward lower scores for VI than for sighted participants occurred for a number of questions describing situations where babble, background noise or reverberation were present, as well as for concentration and effort, but the differences between groups were not significant. The small and non-significant effects may indicate that the VI participants could still make some use of visual cues. Simon and Levitt (2007) proposed that visual impairment would lead to increased difficulty with speech-reading. Indeed, they proposed that even the relatively mild visual impairment resulting from the loss of visual contrast sensitivity with increasing age (Brabyn et al., 2001) may lead to a reduced ability to speech read, since the human face and lips often have relatively low contrast. However, even when the visual image is blurred visual information can still enhance the identification of speech, although the benefit of visual information does decrease with increasing blurring (Thomas and Jordan, 2002). This makes it likely that the VI participants were able to make some use of visual information. The scores for normally sighted participants on the SSQvi questions that were modified from the SSQ questions to remove the visual element (Table 3) suggest that vision did not play a prominent role for the speech questions, but it may have played a role for the spatial questions. The VI and control participants in the current study may have partially based some of their responses to spatial questions on a combination of their auditory and visual abilities. Caution is therefore needed in interpreting the findings. It is possible that people with full blindness would give significantly lower scores for the speech questions about difficult listening situations. The current version of the SSQvi is intended to be applicable to both VI and fully blind participants, and visual cues would not be relevant for blind participants. In future work, it would be desirable to modify some of the questions in the speech section of the SSQvi to specifically assess listening experiences under conditions where visual cues are or are not available, for both VI and normally sighted participants.

For blind participants, age of onset has been shown to affect the extent of sensory compensation, degree of plasticity, and which visual brain regions are recruited for auditory processing (Voss et al., 2008). In the VI group, descriptive information showed that participants with severe visual impairment overall scored lower than those with moderate visual impairment or blindness with remaining vision (Table 6). However, only 2 participants were blind with remaining vision, and further work is need to investigate the effects of severity of visual loss on self-reported auditory abilities. Scores were similar irrespective of both the age of onset (Table 8) and the duration of visual loss (Table 9). Age-related increases in perceived difficulty have been reported for SSQ questions for both normally hearing and sighted individuals, comparing younger (mean age 19 years) to older groups (mean age 70 years) (Banh et al., 2012). Although substantial changes in average scores were not apparent across age groups, the older subgroup of participants aged 41–55 years generally scored higher overall than the younger subgroup aged 30–40 years (Table 10). However, the overall age difference between subgroups was considerably less than that tested by Banh et al. (2012). Tests with more participants are needed to determine how age of onset and the severity of visual impairment affect self-reported auditory abilities.

A factor analysis to investigate the structure of the original SSQ showed three factors with eigenvalues clearly above chance, that corresponded to the Speech, Spatial, and Qualities sections, labeled “speech understanding,” “spatial perception,” and “clarity, separation, and identification” (Akeroyd et al., 2014). A potential fourth factor termed “effort and concentration,” representing two of the qualities questions, was also reported, with eigenvalues bordering on chance. Overall, these results validate the allocation of the SSQ questions into the Speech, Spatial, and Qualities sections. As highlighted by Gatehouse and Noble (2004) and Akeroyd et al. (2014), factor analysis typically requires sample sizes numbering in the hundreds (Tabachnick and Fidell, 2007; Comrey and Lee, 2013). Hence, a factor analysis of the SSQvi was not carried out. A study that conducted factor analysis of the SSQvi using a larger sample, for the English, Tamil and Hindi language versions, would be informative to confirm that the modifications made to the questions did not affect the validity of the allocation of the questions to the three sections.

In the current study, the distribution of men and women was different between the VI and normally sighted control groups, and was not balanced within groups, which makes it difficult to assess gender effects. One study reported gender-related differences for a monaural elevation localization task (Lewald, 2004). The effect of gender on SSQvi responses warrants further investigation. Another potential limitation is the number of years of education completed, that was significantly greater for the sighted control group than for the VI group. Although education levels were not taken into account during data collection, as all the participants understood the task clearly, making it unlikely that education levels would have a material effect on the results, future studies could investigate whether education levels play a role in SSQvi responses. We are unaware of any previous work investigating how gender or number of years of education completed affect self-reported hearing abilities.

### Implications of the findings for rehabilitation

As described in the Introduction, the results contribute to the goal of setting a benchmark for evaluating individual or group scores, by comparing them with the hearing abilities of VI participants who have similar ages and normal hearing or mild hearing loss. Setting such a benchmark would allow clinicians to assess the success of interventions, or set more realistic goals for interventions (Banh et al., 2012). The results could be used as a reference for scores for patients with more severe hearing or vision losses. The original SSQ can be utilized by clinicians in setting rehabilitative goals for those with hearing impairment (Banh et al., 2012). The SSQvi could similarly be used to set rehabilitative goals for those with visual loss, alone or in combination with hearing loss. This may be particularly applicable for the aging population, among whom the incidence of visual and auditory sensory loss is highest. The results indicate a number of situations which VI participants perceived to be difficult and for which they might benefit from intervention. The scores for questions relating to speech indicated that divided-attention contexts in which multiple streams of speech were present were especially difficult for VI participants, as were following conversations in noise and in echoic environments. In the spatial domain, scenarios involving judgments of elevation or distance were especially difficult for VI participants. For the qualities of hearing section, questions related to effort, concentration and ignoring distracting sounds were rated as most difficult.

Since our sample was relatively small, strong conclusions cannot be drawn. Further studies are needed to compare objective data to the subjective data reported here. As described above, comparisons across groups with normal or residual vision could potentially be affected by the use of visual information in addition to hearing in determining responses for some of the questions. The presence of some ceiling effects could reduce the potential use for future research with the current version of the questionnaire and current participant groups. Nevertheless, self-report offers important insight regarding the types of situations that lead to difficulties for those with visual or auditory sensory loss. Further work will allow the mechanisms underlying these difficulties to be studied in further detail, ultimately leading to patient benefit (Gatehouse and Noble, 2004). The SSQvi extends the original SSQ to include participants with visual as well as auditory losses, and offers a promising tool for assessing interventions for patients with single or dual sensory losses.

## Author contributions

Substantial contributions to the conception or design of the work (AK, SC, BM, and SP); the acquisition, analysis, or interpretation of data for the work (AK, SC, BM, SG, RR, and SP), drafting the work (AK), revising it critically for important intellectual content (AK, SC, BM, SG, RR, and SP); final approval of the version to be published (AK, SC, BM, SG, RR, and SP); agreement to be accountable for all aspects of the work in ensuring that questions related to the accuracy or integrity of any part of the work are appropriately investigated and resolved (AK, SC, BM, SG, RR, and SP).

## Funding

The research was supported by the Vision and Eye Research Unit (VERU), Postgraduate Medical Institute at Anglia Ruskin University, and MRC grant G0701870.

### Conflict of interest statement

The authors declare that the research was conducted in the absence of any commercial or financial relationships that could be construed as a potential conflict of interest.

## Appendix

SSQvi: Speech Spatial Qualities Questionnaire for visually impaired listeners.

### Part 1: speech hearing

1. You are talking with one other person and there is a TV on in the same room. Without turning the TV down, can you follow what the person you're talking to says? 0—Not at all, 10—Perfectly.
2. You are talking with one other person in a quiet, carpeted lounge-room. Can you follow what the other person says? 0—Not at all, 10—Perfectly.
3. You are in a group of about five people, sitting round a table. It is an otherwise quiet place. Can you follow the conversation? 0—Not at all, 10—Perfectly.
4. You are in a group of about five people in a busy restaurant. Can you follow the conversation? 0—Not at all, 10—Perfectly.
5. You are talking with one other person. There is continuous background noise, such as a fan or running water. Can you follow what the person says? 0—Not at all, 10—Perfectly.
6. You are talking to someone in a place where there are a lot of echoes, such as a church or railway terminus building. Can you follow what the other person says? 0—Not at all, 10—Perfectly.
7. Can you have a conversation with someone when another person is speaking whose voice is the same pitch as the person you're talking to? 0—Not at all, 10—Perfectly.
8. Can you have a conversation with someone when another person is speaking whose voice is different in pitch from the person you're talking to? 0—Not at all, 10—Perfectly.
9. You are listening to someone talking to you, while at the same time trying to follow the news on TV. Can you follow what both people are saying? 0—Not at all, 10—Perfectly.
10. You are in conversation with one person in a room where there are many other people talking. Can you follow what the person you are talking to is saying? 0—Not at all, 10—Perfectly.
11. You are with a group and the conversation switches from one person to another. Can you easily follow the conversation without missing the start of what each new speaker is saying? 0—Not at all, 10—Perfectly.
12. Can you easily have a conversation on the telephone? 0—Not at all, 10—Perfectly.
13. You are listening to someone on the telephone and someone next to you starts talking. Can you follow what's being said by both speakers? 0—Not at all, 10—Perfectly.

### Part 2: spatial hearing

14. You are outdoors in an unfamiliar place. You hear someone using a lawnmower. Can you tell right away where the sound is coming from? 0—Not at all, 10—Perfectly.
15. You are sitting around a table or at a meeting with several people. Can you tell where any person is as soon as they start speaking? 0—Not at all, 10—Perfectly.
16. You are sitting in between two people. One of them starts to speak. Can you tell right away whether it is the person on your left or your right? 0—Not at all, 10—Perfectly.
17. You are in an unfamiliar house. It is quiet. You hear a door slam. Can you tell right away where that sound came from? 0—Not at all, 10—Perfectly.
18. You are in the stairwell of a building with floors above and below you. You can hear sounds from another floor. Can you readily tell where the sound is coming from? 0—Not at all, 10—Perfectly.
19. You are outside. A dog barks loudly. Can you tell immediately where it is from the sound? 0—Not at all, 10—Perfectly.
20. You are standing on the footpath of a busy street. Can you hear right away which direction a bus or truck is coming from the sound? 0—Not at all, 10—Perfectly.
21. In the street, can you tell how far away someone is, from the sound of their voice or footsteps? 0—Not at all, 10—Perfectly.
22. Can you tell how far away a bus or a truck is, from the sound? 0—Not at all, 10—Perfectly.
23. Can you tell from the sound which direction a bus or truck is moving, for example, from your left to your right or right to left? 0—Not at all, 10—Perfectly.
24. Can you tell from the sound of their voice or footsteps which direction a person is moving, for example, from your left to your right or right to left? 0—Not at all, 10—Perfectly.
25. Can you tell from their voice or footsteps whether the person is coming toward you or going away? 0—Not at all, 10—Perfectly.
26. Can you tell from the sound whether a bus or truck is coming toward you or going away? 0—Not at all, 10—Perfectly.
27. Do the sounds of things you are able to hear seem to be inside your head rather than out there in the world? 0—Inside my head, 10—Out there.
28. Do the sounds of people or things you hear turn out to be closer than expected when you find out where they are? 0—Much closer, 10—Not closer.
29. Do the sounds of people or things you hear turn out to be further away than expected when you find out where they are? 0—Much further, 10—Not further.
30. Do you have the impression of sounds being exactly where you would expect them to be? 0—Not at all, 10—Perfectly.

### Part 3: qualities of hearing

31. Think of when you hear two things at once, for example, water running into a basin and, at the same time, a radio playing. Do you have the impression of these as sounding separate from each other? 0—Not at all, 10—Perfectly.
32. When you hear more than one sound at a time, do you have the impression that it seems like a single jumbled sound? 0—Jumbled, 10—Not jumbled.
33. You are in a room and there is music on the radio. Someone else in the room is talking. Can you hear the voice as something separate from the music? 0—Not at all, 10—Perfectly.
34. Do you find it easy to recognize different people you know by the sound of each one's voice? 0—Not at all, 10—Perfectly.
35. Do you find it easy to distinguish different pieces of music that you are familiar with? 0—Not at all, 10—Perfectly.
36. Can you tell the difference between different sounds, for example, a car vs. a bus; water boiling in a pot vs. food cooking in a frypan? 0—Not at all, 10—Perfectly.
37. When you listen to music, can you make out which instruments are playing? 0—Not at all, 10—Perfectly.
38. When you listen to music, does it sound clear and natural? 0—Not at all, 10—Perfectly.
39. Do everyday sounds that you can hear easily seem clear to you (not blurred)? 0—Not at all, 10—Perfectly.
40. Do other people's voices sound clear and natural? 0—Not at all, 10—Perfectly.
41. Do everyday sounds that you hear seem to have an artificial or unnatural quality? 0—Unnatural, 10—Natural.
42. Does your own voice sound natural to you? 0—Not at all, 10—Perfectly.
43. Can you easily judge another person's mood from the sound of their voice? 0—Not at all, 10—Perfectly.
44. Do you have to concentrate very much when listening to someone or something? 0—Concentrate hard, 10—Not need to concentrate.
45. Do you have to put in a lot of effort to hear what is being said in conversation with others? 0—Lots of effort, 10—No effort.
46. When you are a passenger in a car can you easily hear what the driver is saying sitting alongside you? 0—Not at all, 10—Perfectly.
47. Can you easily ignore other sounds when trying to listen to something? 0—Not easily ignore, 10—Easily ignore.
